# The effectiveness of simulation education program on shared decision-making attitudes among nurses in Taiwan

**DOI:** 10.1371/journal.pone.0257902

**Published:** 2021-09-28

**Authors:** Mei-Hsiang Lin, Shu-Chuan Lin, Yu-Hsia Lee, Pao-Yu Wang, Hon-Yen Wu, Hsiu-Chin Hsu

**Affiliations:** 1 School of Nursing, National Taipei University of Nursing and Health Sciences, Taipei, Taiwan, R.O.C; 2 MacKay Memorial Hospital, Taipei, Taiwan, R.O.C; 3 Department of Nursing, MacKay Junior College of Medicine, Nursing and Management, New Taipei City, Taiwan, R.O.C; 4 Department of Internal Medicine, Far Eastern Memorial Hospital, New Taipei City, Taiwan, R.O.C; 5 School of Medicine, College of Medicine, National Yang Ming ChiaoTung University, Taipei, Taiwan, R.O.C; 6 Institute of Epidemiology and Preventive Medicine, College of Public Health, National Taiwan University, Taipei, Taiwan, R.O.C; 7 Department of Internal Medicine, National Taiwan University Hospital and College of Medicine, Taipei, Taiwan, R.O.C; 8 Department of Internal Medicine, Graduate Institute of Gerontology and Health Care Management, Chang Gung University of Science and Technology, Chang Gung Memorial Hospital, Taoyan, Taiwan, R.O.C; National Taiwan University of Science and Technology, TAIWAN

## Abstract

**Background:**

Shared decision-making (SDM) is significantly associated with promoting the quality of end-of-life (EOL). The attitude of nurses toward the end of life can affect EOL care, but there are few SDM-related clinical learning programs focused on EOL. In this study, therefore, we evaluated the effectiveness of an EOL-simulation education program on attitudes toward SDM among nurses, using an objective structured clinical examination (OSCE).

**Methods:**

We used a quasi-experimental study design to evaluate nurses working at a medical center in Taiwan. We recruited 100 nurses and assigned them to an experimental group (n = 50) and a control group (n = 50). The experimental group received the SDM attitude (SDMA) cultivation program, and the control group did not. After the intervention, all participants were examined in an OSCE to assess the efficacy of their learning. A *p* value of.05 was considered statistically significant.

**Results:**

The average score of the experimental group was higher than that of the control group in the dimensions “empathic communication” and “mastery learning”, but these differences were not significant. SDMA score is significantly and positively correlated with SDMA global score, standardized patient survey (SPS) score, and SPS global score *(r* = .92, .56, and .50, respectively; p < .01).

**Conclusions:**

Simulations concerning EOL care that incorporate SDM components would be effective for training clinical nurses. This study can serve as a reference for nursing-administration managers who may consider designing SDM-related education programs to improve the quality of clinical nursing care.

## Introduction

Owing to increases in life expectancy, as well as rates of cancer and other noncommunicable diseases, the demand for end-of-life (EOL) palliative care is expected to double over the next 20 years [1]. The process of shared decision-making (SDM) may help patients receiving EOL care to better achieve their wishes [2]. An observational study has revealed that patients who had died in the ICU, it was found that the processes of SDM has been significant association with quality of dying care [3]. However, as far as our knowledge, it is unclear which components of SDM are necessary to achieve these outcomes [4]. There is an important role to be played by nurses in ELO to advance the field of SDM [5]. Attitude influences actions and behaviors; therefore, a nurse’s attitude may affect their communication with palliative-care and terminally ill patients [6,7].

Globally it is recommended in healthcare policy, SDM is also central to international policy promoting community palliative care [8]. However, a dearth of studies have explored the diversity of SDM experience among multi-disciplinary professionals such as community nurses, specialist palliative care nurses or allied healthcare professionals [9]. It is not clear what health care professionals’ attitudes towards SDM presenting in their daily activities. These are important research issues, but the related research is still limited [10]. A knowledgeable and skilled nurse with a positive attitude towards shared decision making can facilitate the shared decision-making process [11]. Regarding to the status of SDM attitude among nurses in Asia, a study of South Korea examined the impact of educational programs on nurses’ attitudes towards SDM, which result showed that educational intervention has a significant impact on the attitude of SDM [12]. Another study investigated the knowledge-attitude-behavior of health care professionals towards SDM in Taiwan, 62% of the respondents are nurses, and the results demonstrated that positive attitudes towards SDM must be promoted [10]. Nurses play when engaging in SDM, and they found that educating the patient, delivering information to the multidisciplinary team, supporting the patient psychologically, managing the patient’s side-effects, and advocating for the patient are among the most important [13]. Therefore, SDM-related clinical learning programs focused on EOL were needed.

Studies have shown that SDM improves patients’ health outcomes, adherence to treatment [14], and patient satisfaction with decisions [15], and also improves the overall quality of care rendered [16]. A recent study found that oncology nurses value their participation and contribution to the SDM process, and they believe that they have some impact on the final decision-making process with regard to cancer treatment [13].

Simulation is an active learning strategy that can assist with incorporating EOL care into the nursing curriculum and improve students’ attitudes toward caring for dying patients [17]. In addition, medical and nursing education that incorporates simulation scenarios executed by standardized patients (SPs) for specific clinical situations can provide a high degree of clinical reality [18]. In objective structured clinical examinations (OSCEs), SPs portray a wide range of patient cases and afford students opportunities to interview and examine a live patient in a simulated, safe, and controlled setting, free of the distractions present in real clinical settings [19]. According Isaacson et al., it is essential to expose nursing staff to SDM in EOL contexts and improve their attitudes toward communication practice and mutual influence. Therefore, it is important to explore nurses’ attitudes towards SDM, especially in the context of EOL care, and to develop strategies to alleviate any communication difficulties between nurses and patients, so as to improve care for dying patients [6]. The study aimed to evaluate the effectiveness of EOL simulation education program on SDM attitude among nurses by using an OSCE.

## Methods

### Study design and sample

We used a quasi-experimental study design and conducted the study at a clinical skills center at a medical center in northern Taiwan. We used convenience sampling, based on public outreach posters, to recruit people who agreed to participate in the study. The study ran from January to May 2020. The inclusion criteria were licensed nursing professionals who had received formal nursing education. The exclusion criteria were a confirmed diagnosis of cancer or depression. The recruitment posters were placed in the conference room of every ward in the medical center for three months. We estimated the necessary sample size using G*Power3.1. With settings of α = .05, power = 0.8, and effect size = 0.25, the estimated required sample size was 82 people. Based on a 20% sample loss rate [20], we needed to recruit approximately 100 people in total. Accordingly, we recruited 100 people who met the inclusion criteria. The participants were divided into two groups (experimental and control) in a 1:1 ratio. The experimental group (n = 50) received the SDM attitude (SDMA) cultivation program intervention, while the control group (n = 50) did not receive the intervention.

**Fig 1:**
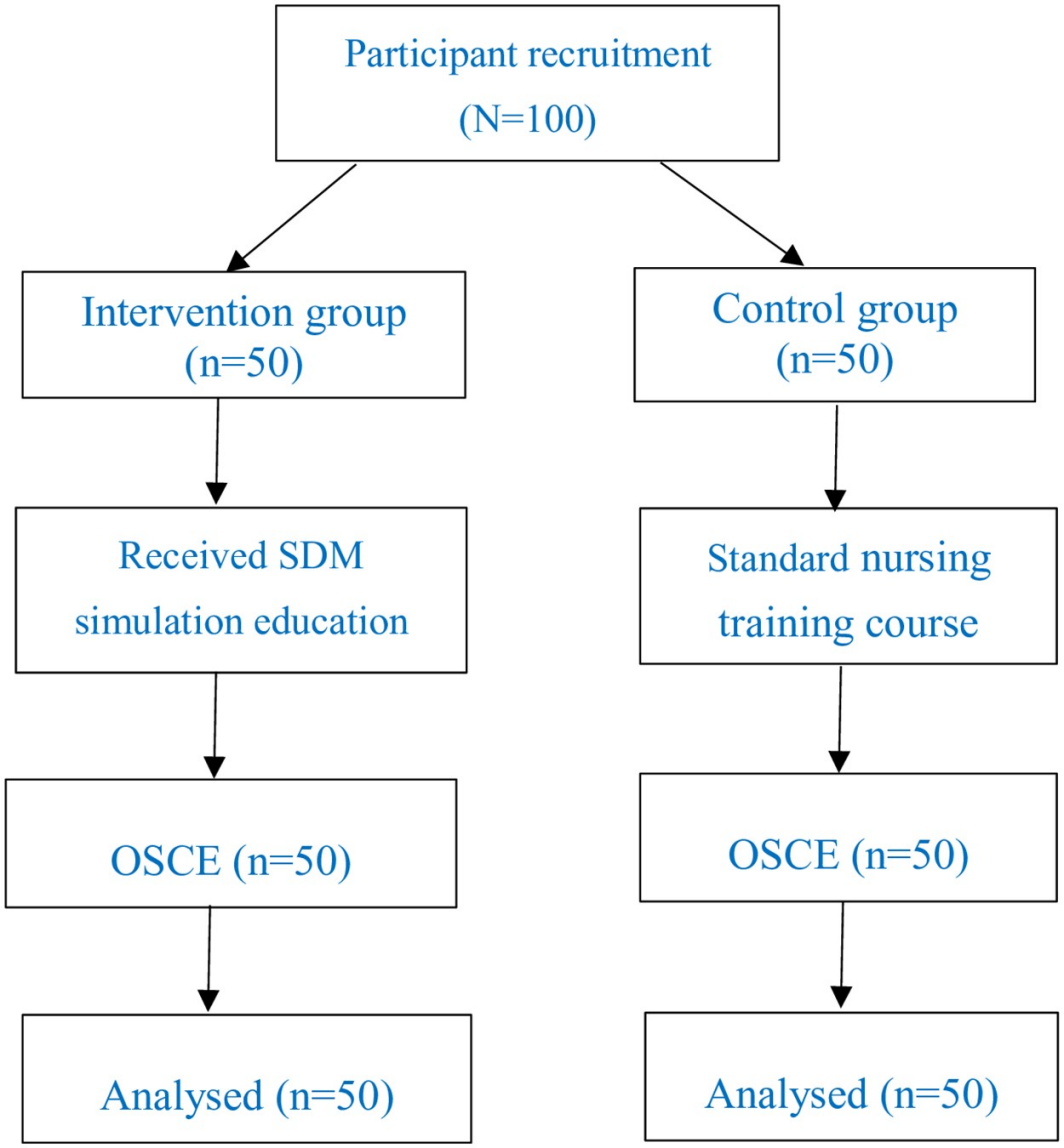
The study flow diagram.

Since there are few existing studies exploring the effectiveness of a simulation-based education program on SDM attitudes among nurses, we selected an effect size of 0.25 based on a study that investigated a cultural-competence cultivation program, the effectiveness of which was examined using an OSCE [21]. The inclusion and exclusion criteria were chosen to reduce the impact of confounding variables. The chart of selection of participants in the study (Fig 1).

#### Instruments

There were no existing measurement tools that could be applied to this research. We therefore developed the instrument for measuring attitude ourselves, based on a literature review of previous qualitative studies [22–24]. We identified EOL-related items that were appropriate to the teaching situation, before classifying and incorporating them into a measurement tool. These measurement tools were then reviewed by five nursing academics for face and content validity. The final versions of these measurement tools are the SDMA scale and the SPS scale (see S1 Appendix for both).

1. **SDMA scale**: This scale was used by the examiners to evaluate the participants’ attitudes during the OSCE. It consisted of an SDMA score, which breaks down attitudes to the SDM process, and an overall performance score that we called the SDMA global score (S1 File).
  **SDMA score**: The assessment comprised 12 questions covering two dimensions: empathic communication and mastery learning. A three-point scale was used: 2 = completely achieved, 1 = partially achieved, 0 = not achieved. The highest possible total score was thus 24, and the higher the total score was, the better was the participant’s attitude toward clinical SDM. Cronbach’s α for this scale was.80.
  **SDMA global score**: The examiner scored the participant’s overall performance in one question during the SDM process while the participant was assisting the patients. A five-point Likert scale was used: 1 = failed, 2 = marginal pass, 3 = average, 4 = good, and 5 = excellent. The examiner assigned the score based on the interaction between the participant and the SP. This questionnaire contained just that one item. The higher the score, the better was the participant’s overall attitude was toward SDM.
2. **SPS scale**: The SPS scale was used by the SP to evaluate the participant’s attitude during the OSCE. It comprised the SPS score and the SPS global score (S2 File).
  **SPS score**: The assessment included five questions on empathy, verbal, and nonverbal communication skills. The SP assigned scores based on their perceptions of interacting with the participant. For each question, the SP assigned two points for “correct”, one point for “partial”, and zero points for “not implemented”. The higher the combined score for the five questions, the better was the nurse’s attitude toward SDM. The Cronbach’s α for this scale was.80.
  **SPS global score**: The SP rated the participant’s overall attitude toward SDM in one question. A four-point Likert scale was used: 1 = failed, 2 = marginal pass, 3 = average, and 4 = excellent.

All SPs participating in this study had completed general SP courses and relevant training regarding the lesson plan and their role in the OSCE. The training totaled eight hours, and included actor readings involving case discussion, profile development, and roleplay to develop, practice, and refine their responses to ensure standardization. The SPs also had a minimum of three hours of experience of actual performance in an OSCE teaching program. Prior to the training course, the investigator discussed the role with the SPs and arranged for the examiners to communicate with them about the plot and to undergo a rehearsal. In this study, the SDMA and SPS scales were examined by five healthcare and education experts who had more than 10 years of clinical experience. Expert validity was tested using the content validity index (CVI) and a four-point scale [25]. The CVI was 1.00 for both research instruments.

*Demographic characteristics*. The demographic characteristics that were recorded were gender, marital status, educational attainment, professional nursing level, department, age, and nursing seniority.

### Procedure

The SDMA cultivation program ran for two weeks and consisted of one session per week comprising three hours of SDM basic-concept lectures and three hours of video-based simulation teaching. The educational intervention, with particular focus on the SDM video simulations that were created for this program, referenced the Three Talk model proposed by Elwyn et al. [8]. The videos were separated into “appropriate” and “inappropriate” versions and covered the following three scenarios. (1) Scenario 1: choice talk that included preliminary instructions regarding the clinical choice of receiving or not receiving intensive care. (2) Scenario 2: option talk that covered the pros and cons of choosing to receive or not receive intensive care, as well as relevant patient considerations and preferences. (3) Scenario 3: decision talk consisting of discussions following the decision on whether or not to receive intensive care.

The simulation sessions were carried out in three phases. In Phase 1, five minutes were spent on refreshing the participants’ experience and managing their expectations, explaining the learning objectives and how the group teaching would be carried out, and introducing the cases. Phase 2 involved immersion, observation, and reflection. The participants watched a 10-minute video (the inappropriate version) about patients faced with a decision regarding EOL care. In the video, the nursing staff used clinical decision-making steps to communicate with family members about whether the patient should receive intensive care. This was followed by a 10-minute instructor-led group discussion. Phase 3 involved reflection, feedback, and conversation. The participants then watched a 20-minute video (the appropriate version), before engaging in 10 minutes of discussion followed by 10 minutes of communication practice. By using a clinical simulation comprising these three phases, the participants were able to engage in case discussions for both the appropriate and the inappropriate scenario. Based on feedback shared by the participants, the choice talk, option talk, and decision talk continued throughout the reflection process until the end of the three phases. All three phases were conducted by two faculty members who were registered nurses with more than five years’ clinical experience in EOL care.

Two months after the education intervention, the two groups of participants were notified by phone and by email about the OSCE assessment. The participants took the test in the order in which they registered for it online, regardless of whether they were in the experimental or the control group. The examiners and SPs were blinded to the group to which the participants belonged. Based on previous research, conducting the OSCE evaluation two months after the intervention would better reflect the long-term effect of the research [26]. This delay prevents the participants from simply imitating the actions of the models in the appropriate video and allows them to experience some actual clinical practice opportunities between the intervention and the test.

The OSCE was carried out based on the steps as follows [27]:

**Teaching plan, writing, and editing**: Practical cases that had teaching significance in a clinical setting were selected to assist with the design of teaching plans and SP scripts. The content included examinee instructions, examiner instructions, SP instructions, and score sheets.
**Examiner consensus**: Before the actual OSCE, Cronbach’s α was found to be.90 for the examiners’ joint evaluation of the training videos, implying acceptable consistency among the examiners.
**OSCE operation process**:
  1 The simulation content was posted on a bulletin board at the door. The scenario concerned a Ms. Wang, a 60-year-old female who had lived in the United States for a long time and had returned home because her 78-year-old mother had developed stage IV breast cancer and undergone six chemotherapy sessions. However, the cancer had metastasized to her lungs and her brain, and she was now unconscious. Ms. Wang’s mother had developed sighing breathing and had occasional 10–30-second pauses in her breathing. Doctors had explained to her family members that the disease could no longer be actively treated. Faced with these changes in her mother’s condition, Ms. Wang looked worried and helpless at the nursing station. She was psychologically unable to accept the sudden and unexpected situation. She had hoped to be able to do something more for her mother. She felt regretful about her mother’s condition and wanted to consult the nursing staff about whether her mother should receive intensive care.
  2 The examinee instructions included relevant details such as background information, test theme, and test time.
  3 Operational content: The participant was expected to use SDM tools to explain the options of receiving or not receiving intensive care to the patient, who was suffering from an EOL condition. During the OSCE process, the examiners used the SDMA scale to evaluate the participant. Additionally, the SP evaluated the participant’s performance using a SPS scale after the OSCE process.

### Data analysis

Data processing and analysis was performed using SPSS 22.0 for Windows. Descriptive statistics were used to calculate the average values of the variables; differences between groups were compared via independent sample *t*-tests, and associations among the OSCE assessment instruments were determined via Spearman correlation analysis. The homogeneity of the two groups was evaluated via a chi-squared test, an independent samples *t*-test, or a Fisher’s exact test. The dependent variables were the effectiveness of EOL simulation education program on SDM attitude by using an OSCE. The SDMA cultivation program was the independent variables (S3 File).

#### Ethical considerations

This study was approved by the Ethical Review Committee of the Mackay Memorial Hospital Institutional Review Board (approval No. 19MMHIS154e). Prior to the experiment, the study objectives and data collection procedures were fully explained to the study participants. In addition, participants were informed that they could withdraw at any time during the process if they did not feel well or no longer wanted to participate. Data collected were filed anonymously, and the participants signed a written consent form before the beginning of the experiment.

## Results

### Characteristics of the participants

Most of the study participants were female (n = 99). The average age of the participants was 37.36 years (standard deviation [SD] = 10.45 years). The average age of the experimental group (34.86 ± 10.42 years) was slightly lower than that of the control group (39.86 ± 9.96 years). Most of the participants were college graduates (72 people, 72.0%) with an average work experience of 15.65 years (SD = 10.87 years). The numbers of participants working in the internal medicine ward, surgical ward, intensive care unit, outpatient department, and pediatric ward were 22 (22.0%), 19 (19.0%), 19 (19.0%), 16 (16.0%), and 11 (11.0%), respectively. The average number of years that the experimental-group participants had worked (13.14 ± 10.70 years) was slightly longer than that of the control group (18.15 ± 10.57 years) (Table 1). There was no significant difference in gender, marital status, or the highest level of education between the two groups (*p* >.05).

**Table 1:** Participant characteristics in the two groups.

Variables	Total (*N* = 100)	Experimental group (n = 50)	Control group (n = 50)	χ^2^ value	*P* value
**Gender** ^c^					1.000
Male	1 (1.0%)	1 (2.0%)	0 (0.0%)		
Female	99 (99.0%)	49 (98.0%)	50 (100.0%)		
**Marital status** ^a^				0.36	.546
Married	45 (45.0%)	24 (48.0%)	21 (42.0%)		
Unmarried	55 (55.0%)	26 (52.0%)	29 (58.0%)		
**Educational attainment** ^a^				0.26	.880
Diploma	20 (20.0%)	11 (22.0%)	9 (18.0%)		
Bachelor’s degree	72 (72.0%)	35 (70.0%)	37 (74.0%)		
Master’s degree	8 (8.0%)	4 (8.0%)	4 (8.0%)		
**professional nursing level** ^c^				12.33	.012
N0	7 (7.0%)	5 (10.0%)	2 (4.0%)		
N1	25 (25.0%)	17 (34.0%)	8 (16.0%)		
N2	24 (24.0%)	13 (26.0%)	11 (22.0%)		
N3	38 (38.0%)	15 (30.0%)	23 (46.0%)		
N4	6 (6.0%)	0 (0.0%)	6 (12.0%)		
**Department** ^c^				14.84	.027
Internal medicine ward	22 (22.0%)	11 (22.0%)	11 (22.0%)		
surgical ward	19 (19.0%)	10 (20.0%)	9 (18.0%)		
gynaecological ward	5 (5.0%)	0 (0.0%)	5 (10.0%)		
paediatric ward	11 (11.0%)	6 (12.0%)	5 (10.0%)		
emergency department	5 (5.0%)	0 (0.0%)	5 (10.0%)		
intensive care unit	19 (19.0%)	9 (18.0%)	10 (20.0%)		
haemodialysis department	3 (3.0%)	3 (6.0%)	0 (0.0%)		
outpatient department	16 (16.0%)	11 (22.0%)	5 (10.0%)		
	***Mean ± SD***	***Mean ± SD***	***Mean ± SD***	***t* value**	***P* value**
**Age**(year) ^b^	37.36 ± 10.45	34.86 ± 10.42	39.86 ± 9.96	-2.45	.016
**Nursing seniority** (year) ^b^	15.65 ± 10.87	13.14 ± 10.70	18.15 ± 10.57	-2.36	.020

Note:

^a^ Chi-square test;

^b^ Independent student t-test;

^c^ Fisher’s exact test.

### The SDMA cultivation program effectiveness

The objective of this study was to evaluate the effectiveness of EOL simulation education on nurses’ attitudes toward SDM by using an OSCE. The average SDMA score for the experimental group was 17.78 (SD = 3.58), which was slightly higher than that of the control group (17.62 ± 3.51), although this difference was not statistically significant (*p* = .82; Table 2). The experimental group also had a higher average SDMA global score (3.26 ± 1.42) than the control group (3.10 ± 1.21), but this difference was also not statistically significant (*p* = .54). For the empathic communication dimension, the experimental group obtained a higher average score for the items “Shows empathy (listens to the inquirer attentively without interrupting)” and “Avoids judgmental words and attitudes towards the inquirer” than the control group, but these differences were not statistically significant (*t* = 1.54, *p* = .12; *t* = 1.00, *p* = .30). For the mastery learning dimension, the experimental group obtained a higher average score for the item “Be able to make a suggestion if the inquirer is unable to choose between the options” than the control group, but again the difference was not statistically significant (*t* = 1.38, *p* = .16).

**Table 2:** Intergroup differential analysis of the OSCE scores of the two groups.

Variables	Experimental group		Control group		t	*p*
	Mean	SD	Mean	SD		
**Empathic communication**	8.58	1.48	8.54	1.64	.12	.89
1.Shows respect to the inquirer (eye contact and tone) with a sincere attitude	1.92	.34	1.94	.23	-.34	.73
2.Shows empathy (listens to the inquirer attentively without interrupting)	1.78	.41	1.62	.60	1.54	.12
3.Comforts the inquirer and gives timely emotional support	1.24	.77	1.28	.700	-.27	.78
4.Responds and confirms (responds to the inquirer’s question in a timely manner and checks the inquirer understands what was said)	1.64	.48	1.72	.49	-.81	.41
5.Avoids judgmental words and attitudes towards the inquirer	2.00	.00	1.98	.14	1.00	.32
**Mastery learning**	9.20	2.52	9.08	2.46	.24	.81
6.Be able to use SDM tools (such as health education booklets, videos, PDAs)	1.68	.55	1.82	.38	-1.46	.14
7.Guide the inquirer to talk about factors the inquirer cares about (such as economics, quality of life, risks, sequelae…) when choosing a treatment plan and their importance	1.58	.60	1.66	.47	-.73	.46
8.Be able to assist the inquirer in confirming the preliminary decision of the option	1.48	.73	1.48	.70	.00	1.00
9.Assess and confirm the inquirer’s awareness about the choice of the options	1.64	.59	1.66	.59	-.16	.86
10.Be able to confirm the inquirer’s intended option	1.24	.82	1.04	.78	1.24	.21
11.Be able to make a suggestion if the inquirer is unable to confirm their choice between the options	1.42	.60	1.24	.68	1.38	.16
12.Complete the shared decision-making aid evaluation form	.18	.52	.18	.56	.00	1.00
SDMA score	17.78	3.58	17.62	3.51	.22	.82
SDMA global score	3.26	1.42	3.10	1.21	.60	.54

SDMA: Shared Decision-Making Attitude; PDAs: Patient decision aids.

The experimental group obtained a slightly higher average SPS score (8.06; SD = 1.78) than the control group, but the difference was not statistically significant (*p* = .78; Table 3). The average SPS global score for the experimental group (3.36 ±.56) was also slightly higher than that of the control group (3.34 ±.59), although this difference was also not significant (*p* = .86). The SDMA score was positively correlated with the SDMA global score (*r* = .92; *p* < .01; Table 4). The SDMA score was also positively correlated with the SPS score and the SPS global score (*r* = .56, *r* = .56; *p* < .01), as was the SDMA global score (*r* = .54, *r* = .52; *p* < .01).

**Table 3:** The between-group comparisons of SPS scores.

items	Experimental group		Control group		t	p
	mean	SD	mean	SD		
1. The nurse listened to what I said and used words that I understand.	1.80	.40	1.86	.35	-.79	.43
2. The nurse responded to me appropriately and empathetically.	1.50	.61	1.40	.63	.79	.42
3. The nurse understood the situation and did not speak too fast.	1.84	.37	1.74	.44	1.22	.22
4. The nurse calmed me down appropriately when I was emotional.	1.20	.72	1.22	.81	-.12	.89
5. The nurse was able to use SDM tools to solve my problems appropriately.	1.72	.49	1.74	.44	-.21	.83
SPS score	8.06	1.78	7.96	1.87	.27	.78
SPS global score	3.36	.56	3.34	.59	.17	.86

SPS: Standardized Patient Survey.

**Table 4:** The associations among various OSCE assessment tools.

variables	1	2	3	4
1. SDMA score	1			
2. SDMA global score	.92**	1		
3. SPS score	.56**	.54**	1	
4. SPS global score	.50**	.52**	.77**	1

SPS: Standardized Patient Survey; SDMA: Shared Decision-Making Attitude;

** p < .01.

## Discussion

The study aimed to determine the effects of EOL simulation education program on SDM attitude among nurses by using an OSCE. The results showed that the SDMA score did not reach a significant difference between the two groups. This finding was contradictory with a previous study [28], which found that EOL care simulation using SPs was an effective strategy for training nursing students who had limited opportunities to experience EOL care.

Two reasons might explain this phenomenon. First, the SDM being an accredited skill for hospitals in Taiwan, with medical institutions holding SDM-related on-service training that can affect nursing staff’s SDM attitude. Secondly, the average age and work experience of the experimental group were less than control group in this study. The age and work experience of nursing staff affected the quality of the care they provided [12,29]. At this point, those potential confounders should be taken in account to influencing the results. Future research might conduct stratified randomized sampling based on age and nursing seniority. Nevertheless, the SDMA score of the experimental group was better than that of the control group in this study. The finding was echoed a previous study, that it is crucial for nurses learn the required attitudes of nursing through training as part of their socialization into the profession [30]. Additionally, as the nursing staff is familiar with the concept of SDM will agree and be willing to implement SDM [31]. Therefore, the present study showed that the SDMA score of the experimental group was better than that of the control group.

The main goal of the OSCE was to evaluate the clinical practice skills of the candidates, such as their ability to understand a patient’s experience of their condition, and their ability to understand each patient as a whole [32]. Both groups of participants were examined via the OSCE to assess the efficacy of the video-based scenario simulation intervention. Our findings were consistent with those of Waschwill et al. [33], who used scenario simulation to evaluate medical students’ SDM skills. They also found that those who had not participated in the SDM training could also complete the tasks. Our results were also similar to those of Lee et al. [21]. In their study, the experimental group received a cultural-competence cultivation program and the control group did not receive any interventions. Both groups completed the OSCE assessment after the intervention, and although the average score of the experimental group was slightly higher than that of the control group, the difference was not significant [21].

A recent study reported that effective communication, joint decision-making, and assessment of the needs of the patient and other important people in the decision-making process, are important goals in medical practice [34], and our findings are congruent with this. The average scores for the items “shows empathy (listens to the inquirer attentively without interrupting)” and “avoids judgmental words and attitudes toward the inquirer” were higher for the experimental group than for the control group. This echoed the findings of Isaacson et al. [9] that nurses must be skillful and empathetic communicators. Importantly, the fact that our findings agreed with those of previous studies implies that simulation is an effective active learning strategy. Further, specialized EOL care training should include an EOL communication model accompanied by experiential learning, debriefing, and an emphasis on the mutual influence of patient, family, and nurses [9,17]. A recent study compared single and multiple learners undergoing standardized training in patient communication skills for palliative care [27]. It demonstrated that the learners’ comfort with the skill set increased significantly during an SP simulation that involved using communication skills to deliver difficult news. In our study, the average SPS score and SPS global score for the experimental group were slightly higher than those of the control group, but the difference was not statistically significant. The SPs were the direct recipients of the communication by the participants during the OSCE assessment, so their evaluation directly reflects the participants’ communication skills. Although the difference between the two groups was not statistically significant, the results of this study can nevertheless be used as a clinical reference. A measure of content validity was used to test the OSCE score scales, and it was found to be 1.00. A previous study found that when three to five experts are interviewed, the CVI should ideally be 1.00 [25], confirming that the content validity of the scales used in our study was acceptable.

There was a significant correlation between the SDMA scores and the SDMA global score, and also between the SPS scores and the SPS global score. These findings are consistent with several previous studies. Yedidia et al. [35] conducted an OSCE assessment to evaluate the communication skills of third-year medical students from three different medical schools. As in the present study, examiners and SPs were engaged to evaluate the students and underwent standardized training and assessment to ensure case scenario fidelity. In the current study, the inclusion of SPs in the OSCE process supported the statement by Miller et al. [18] that the SP executes specific clinical situations, providing learners with a high degree of clinical reality. Ndiwane et al. [36] also included an SP when conducting their OSCE assessment of cultural competence among first-year nursing students. They found that the examiner’s score and the SP’s assessment score were significantly correlated. The participants also confirmed that the SP’s score was objective. These findings suggest that the examiner’s evaluation of the participants’ performance is positively correlated with the SP’s perception and is thus a valid assessment of a nurse’s performance.

Nurses’ attitudes toward EOL patient care may depend on the department in which they work in their clinical practice [6]. The sample used in the present study was recruited from an intensive care unit, an outpatient department, and medical, surgical, and pediatric wards. Our results revealed a statistically significant difference between both groups in the departments. This is also highlighted in other research. For example, Spear [23] showed that the attitudes of nurses providing EOL care could be influenced by their demographic characteristics, experience, and previous education. We did not address these factors here, which was a limitation of this study. In the future, we should seek to better understand nurses’ attitudes toward caring for dying patients, conduct a homogeneity test of EOL care, understand the factors influencing EOL care, and ensure that the study groups are homogeneous prior to the intervention.

### Limitation

We did not conduct an OSCE assessment before the intervention based on the consideration that the initial test may affect the inherent validity of the post-intervention test results. In addition, nowadays SDM being an accredited skill for hospitals in Taiwan, with medical institutions holding SDM-related on-service training that can affect nursing staff’s SDM attitude. Moreover, we also did not collect any information about the participants’ attitudes toward SDM before the intervention, which limited our ability to draw inferences from the results. In addition, nurses who already had a positive attitude toward SDM and EOL care may have been more inclined to participate in this study. Therefore, the possibility of bias in the sample selection process cannot be ruled out. Future studies should expand their sample sources to explore the attitudes toward SDM and EOL care of a wider range of nursing staff, thus providing a more complete reference base for relevant patient care. Finally, there was the potential for cross-contamination, since the participants were recruited from a single hospital. Future studies should consider randomly selecting participants from different hospitals, which would help to avoid cross-contamination.

## Conclusion

Although the SDM attitude score of the experimental group was higher than that of the control group, the SDM situational simulation program had no significant effect on the nurses’ attitudes toward SDM. However, such attitudes form an essential point of consideration in patient-centered care. This evaluation provided empirical evidence that simulating EOL care with SDM could be an effective strategy for training clinical nurses and improving their attitudes toward SDM. It thus provides a reference for designing a clinical nursing curriculum to incorporate SDM and thus ensure a better standard of EOL nursing care for patients.

## Supporting information

S1 File SDMA scale. (DOCX)

S2 File SPS scale. (DOCX)

S3 File SDM-OSCE dataset. (SAV)

S1 Appendix (DOCX)
