# Supplementary material for: The effectiveness of simulation education program on shared decision-making attitudes among nurses in Taiwan
Source: PLoS One. 2021 Sep 28;16(9):e0257902. doi: 10.1371/journal.pone.0257902 (PMC8478250; doi:10.1371/journal.pone.0257902)
Supplement: S2 File — (DOCX) [file pone.0257902.s002.docx]

**S2 File. SPS scale**

**SPS- for SP evaluation**

| items | | | | | Total scores： | | | |
| --- | --- | --- | --- | --- | --- | --- | --- | --- |
|  |  |  |  |  | Correct (2) | Partial (1) | Not implemented (0) | Remarks |
| 1. The nurse listened to what I said and used words that I understand. | | | | |  |  |  |  |
| 2. The nurse responded to me appropriately and empathetically. | | | | |  |  |  |  |
| 3. The nurse understood the situation and did not speak too fast. | | | | |  |  |  |  |
| 4. The nurse calmed me down appropriately when I was emotional. | | | | |  |  |  |  |
| 5. The nurse was able to use SDM tools to solve my problems appropriately. | | | | |  |  |  |  |
| **Overall performance** | excellent | average | marginal pass | failed |  | | | |
|  |  |  |  |  |  |  |  |  |
