# Supplementary material for: The effectiveness of simulation education program on shared decision-making attitudes among nurses in Taiwan
Source: PLoS One. 2021 Sep 28;16(9):e0257902. doi: 10.1371/journal.pone.0257902 (PMC8478250; doi:10.1371/journal.pone.0257902)
Supplement: S1 Appendix — (DOCX) [file pone.0257902.s004.docx]

**Appendix.**

**(一) SDMA- for examiner assessment**

| **items** | completely achieved (2) | Partially achieved (1) | Not achieved (0) | Remarks |
| --- | --- | --- | --- | --- |
| 1.Shows respect to the inquirer (eye contact and tone) with a sincere attitude |  |  |  |  |
| 2.Shows empathy (listens to the inquirer attentively without interrupting) |  |  |  |  |
| 3.Comforts the inquirer and gives timely emotional support |  |  |  |  |
| 4.Responds and confirms (responds to the inquirer’s question in a timely manner and checks the inquirer understands what was said) |  |  |  |  |
| 5.Avoids judgmental words and attitudes towards the inquirer |  |  |  |  |
| 6.Be able to use SDM tools (such as health education booklets, videos, PDAs) |  |  |  |  |
| 7.Guide the inquirer to talk about factors the inquirer cares about (such as economics, quality of life, risks, sequelae...) when choosing a treatment plan and their importance |  |  |  |  |
| 8.Be able to assist the inquirer in confirming the preliminary decision of the option |  |  |  |  |
| 9.Assess and confirm the inquirer's awareness about the choice of the options |  |  |  |  |
| 10.Be able to confirm the inquirer’s intended option |  |  |  |  |
| 11.Be able to make a suggestion if the inquirer is unable to confirm their choice between the options |  |  |  |  |
| **Total scores:** | □ pass □ Failed | | | |
| \| Global Score \| excellent  (5) \| good  (4) \| average  (3) \| marginal pass  (2) \| failed  (1) \| Remarks \| \| --- \| --- \| --- \| --- \| --- \| --- \| --- \| \|  \|  \|  \|  \|  \|  \| | | | | |

**(二) SPS- for SP evaluation**

| items | | | | | Total scores： | | | |
| --- | --- | --- | --- | --- | --- | --- | --- | --- |
|  |  |  |  |  | Correct (2) | Partial (1) | Not implemented (0) | Remarks |
| 1. The nurse listened to what I said and used words that I understand. | | | | |  |  |  |  |
| 2. The nurse responded to me appropriately and empathetically. | | | | |  |  |  |  |
| 3. The nurse understood the situation and did not speak too fast. | | | | |  |  |  |  |
| 4. The nurse calmed me down appropriately when I was emotional. | | | | |  |  |  |  |
| 5. The nurse was able to use SDM tools to solve my problems appropriately. | | | | |  |  |  |  |
| Global Score | excellent | average | marginal pass | failed |  | | | |
|  |  |  |  |  |  |  |  |  |
